# Perceived Stress among Undergraduate Students in a Dental College: A Descriptive Cross-sectional Study

**DOI:** 10.31729/jnma.6446

**Published:** 2021-09-30

**Authors:** Sunita Khanal, Sujita Shrestha

**Affiliations:** 1 Department of Community Dentistry, Kantipur Dentai College, Basundhara, Kathmandu, Nepal

**Keywords:** *dental students*, *prevalence*, *stress*

## Abstract

**Introduction:**

An individual may feel stressed when he/she is unable to cope with past, present, and future situations. A high level of stress in dental students is due to the fact that dental students need to acquire diverse proficiencies such as theoretical knowledge, clinical competencies, and interpersonal skills. So, this study was conducted to find out the prevalence of perceived stress among undergraduate students in a dental college.

**Methods:**

A descriptive cross-sectional study was conducted among first-year to final year students of a tertiary care centre from July 2020 to August 2020. The sample size was calculated to be 177. Convenience sampling technique was used. Data collection was done after taking ethical approval from the institutional review committee (Ref no.21/020). Data collection was done by using a selfadministered, modified Dental Environmental Stress Questionnaire. Data analysis was done by using Statistical Package of Social Sciences version 20 software. Point estimate at 95% Confidence Interval was calculated along with frequency and proportion for binary data.

**Results:**

The prevalence of perceived stress was found to be 80.55 (45%) at 95% Confidence Interval (37.71-52.28). Students perceived more stress from the domain related to personal factors i.e. fear of failing 78 (43.6%), academic factors i.e. examination and grades 71 (39.7%), unable to catch up with the back lock work 68 (38%), and for the clinical phase students, patients not coming or coming too late was most stressful 69 (60%) followed by getting an ideal case for clinical examination 60 (52.2%).

**Conclusions:**

Overall stress levels were slight to severe and were comparatively higher in clinical phase students due to factors related to patients.

## INTRODUCTION

Stress can be defined as the pressure accompanying a perceived demand to be challenging or threatening depending upon evaluation.^1^ Any external demand on an individual's psychological and physical well-being is stress.^2^

Worldwide, dental training is perceived to be a very challenging and stressful experience by the great professionals where the students have to undergo expansive preclinical, clinical, and interpersonal skills as well as training.^3^ Many studies have been done globally to assess the prevalence of stress among medical students and it was found to be 25-90% from beginning till their graduation.^4^ Very few studies have been done in Nepal regarding the perceived stress among undergraduate dental students.

So, the main aim of this study is to find out the prevalence of perceived stress among undergraduate dental students.

## METHODS

A descriptive cross-sectional study was conducted among all the students of Kantipur Dental College. The data was collected from the first year to final year students from July 2020 to August 2020. The data was collected via a web-based online survey by sending the link of the semi-structured questionnaire in the form of a Google document.

Ethical approval was taken from the institutional review committee of Kantipur Dental College (Ref no.21/020). Before the commencement of the study, objectives were clearly explained to the students, and consent was obtained from them. All the students from the first year to final year who were interested to participate in the study were included in the study. Exclusion criteria included those students who were not willing to participate in the study.

A convenience sampling technique was done.

Sample size calculation was done by using the following formula.

Sample size n = Z^2^ × p × q / e^2^

    = (1.96)^2^ × 0.341 × 0.659 / (0.07)^2^

    = 177

where,

n = required sample size,
Z = 1.96 at 95% Confidence Interval
p = prevalence taken as 34.1%^5^
q = 1-p
e = margin of error, 7%

Data collection was done by using self-administered, modified Dental Environmental Stress (DES) Questionnaire.^1^ Pretesting was done. Only 21 questions that are applicable in our context were included and they were further divided into four domains:- i) Stress due to academic factors, ii) Stress associated with faculty, iii) personal stress, and iv) stress due to clinical factors. The fourth domain was for clinical phase students i.e. third year to final year students only. One to three domains were for all students but the fourth domain was only for clinical students. The response for each question was based on a Likert-type scale with response options of 1-not stressful, 2-slightly stressful, 3-moderately stressful, and 4-severely stressful.

The questionnaire had demographic details in the first section and possible sources of stress in the second section. The questionnaire was distributed to the participants through their email addresses, Viber, and Facebook using google forms. The data collected via the online survey were imported to an excel sheet, cleaned, and analysed in Statistical Package for Social Sciences version 20. Descriptive parameters such as frequency table and the percentage were calculated and tabulated.

## RESULTS

The prevalence of perceived stress was found to be 80.55 (45%) at 95% Confidence Interval (37.71-52.28).

Altogether 179 students participated in the study among which 31 (17.3%) were male and 148 (82.7%) were female (Table 1).

**Table 1:** Showing the total distribution of the participants.

Phase		Preclinical		Clinical			Total n (%)
Year of study		1^st^ n (%)	2^nd^ n (%)	3^rd^ n (%)	4^th^ n (%)	Final n (%)	
Gender	Male	1 (3.1)	2 (6.2)	9 (26.5))	7 (20)	12 (26.1)	31 (17.3)
	Female	31 (96.9)	30 (93.8)	25 (73.5)	28 (80)	34 (73.9)	148 (82.7)
No. of participants		32 (17.87)	32 (17.87)	34 (19.00)	35 (19.55)	46 (25.69)	179
Total		64 (35.75)		115 (64.24)			179

In the first domain i.e. stress due to academic factors; the amount of assigned work 88 (49.10%), competition within the batch mates 81 (45.5%), and difficulty in learning and submitting assignments on time (36.40%) was slightly stressful whereas examination and grades 71 (39.70%), allocated time to complete practical 66 (36.90%) and unable to catch up with back lock work 68 (38%) was severely stressful (Table 2).

**Table 2:** Showing stress level due to academic factors.

	Not stressful n (%)	Slightly stressful n (%)	Moderately stressful n (%)	Severely stressful n (%)
Amount of assigned work	32 (17.90)	88 (49.10)	43 (24.10)	16 (8.90)
Competition within batch mates	47 (26.30)	81 (45.30)	46 (25.70)	5 (2.80)
Examination and grades	9 (5.10)	39 (21.80)	60 (33.50)	71 (39.70)
Difficulty in learning and submitting assignments on time	17 (9.50)	65 (36.40)	53 (29.60)	44 (24.60)
Allocated time to complete practical	13 (7.30)	51 (28.50)	50 (28)	66 (36.90)
Unable to catch up with back lock work	12 (6.70)	52 (29.10)	47 (26.30)	68 (38)

In the second domain i.e. stress associated with faculty: criticism from faculty 51 (28.5%) was moderately stressful, biased nature of faculty 49 (27.50%) was severely stressful, the atmosphere created by faculty in the department 53 (29.60%), approachability of faculty 68 (38.10%) and different opinion of faculty 60 (33.60%) were slightly stressful but different rules of different faculty 60 (33.60%) was not stressful (Table 3).

**Table 3:** Showing stress level associated with faculty.

	Not stressful n (%)	Slightly stressful n (%)	Moderately stressful n (%)	Severely stressful n (%)
Criticism from faculty	35 (19.60)	49 (27.50)	51 (28.50)	42 (23.50)
Biased nature of faculty	39 (21.80)	49 (27.40)	42 (23.50)	49 (27.50)
Atmosphere created by faculty in the department	49 (27.40)	53 (29.60)	48 (26.80)	29 (16.20)
Different rules of different faculty	60 (33.50)	53 (29.70)	46 (25.70)	20 (11.20)
Approachability of faculty	42 (23.50)	68 (38.10)	43 (24)	25 (14)
Different opinions of faculty	40 (22.30)	60 (33.60)	44 (24.60)	35 (19.60)

In the third domain i.e. personal stress; relation with batch mates 115 (64.2%) and atmosphere at home/hostel 69 (38.5%) was not stressful, fear of failing 78 (43.6%) and fear of facing parents after results 54 (30.2%) were severely stressful but financial burden 54 (30.2%) was slightly stressful (Table 4).

**Table 4:** Showing stress level due to personal factors.

	Not stressful n (%)	Slightly stressful n (%)	Moderately stressful n (%)	Severely stressful n (%)
Relations with batchmates	115 (64.2)	46 (25.7)	14 (7.8)	4 (2.2)
Atmosphere at hostel/home	69 (38.5)	55 (30.7)	28 (15.6)	27 (15.1)
Fear of failing	16 (8.9)	36 (20.1)	49 (27.4)	78 (43.6)
Fear of facing parents after results	33 (18.4)	47 (26.3)	45 (25.1)	54 (30.2)
Financial burden	36 (20.1)	54 (30.2)	50 (27.9)	39 (21.8)

In the fourth domain i.e. stress due to clinical factors; lack of co-operation by patients 43 (37.4%), Patients coming late or not coming for appointments 69 (60%) and getting an ideal case for clinical examination 60 (52.2%) was severely stressful but lack of confidence in clinical decision making 38 (33%) was moderately stressful (Table 5).

**Table 5:** Showing stress level due to clinical factors.

	Not stressful n (%)	Slightly stressful n (%)	Moderately stressful n (%)	Severely stressful n (%)
Lack of co-operation by patients	8(7.0)	25 (21.7)	39 (33.9)	43 (37.4)
Patients coming late or not coming for appointments	4(3.5)	19 (16.5)	23 (20)	69 (60)
lack of confidence in clinical decision making	11 (9.6)	37 (32.2)	38 (33)	29 (25.2)
Getting an ideal case for clinical examination	10 (8.7)	21 (18.3)	3 (20.9)	60 (52.2)

## DISCUSSION

The main aim of the dental training program is to produce competent graduates who will provide quality dental services to their communities. During the training program, the students have to spend many hours on lectures and practicals. Apart from that, students keep on trying their best to achieve the expected academic outcomes which may lead to stress.

In the present study, the majority of the students found allocated time to complete practical and unable to catch up with back lock work to be severely stressful which is similar to other studies.^6-7^ This may be because of the intensive amount of academic work as well as clinical and preclinical exercises.

Criticism from faculty was moderately stressful, biased nature of faculty was severely stressful whereas atmosphere created by faculty in the department, approachability of faculty and different opinion of faculty were slightly stressful which is in accordance to other studies.^1,7-8^ Some students feel stressed if criticized in front of patients and friends. The tendency of giving priority to students who secure good grades is also stressful for the students. Therefore, a change concerning the attitude of staff towards the academic environment where examination and grades are given main importance needs to be altered. Also, sufficient faculties should be employed in each department so that when one faculty is not available the students can benefit from other faculties.

Personal stress was found to be the most stressful for the students. In this domain fear of failing and fear of facing parents after results were severely stressful. This finding is similar to other studies.^7,9,10^ This may be because, despite their hard efforts, students find it very difficult to predict their results. Many students are not able to clear dentistry and even drop the course which is very painful for them and family.

But the financial burden was slightly stressful which is similar to other studies.^9,11^ But in contrast to another studies.^10,12^ This can be because of the fact that admission to dental college requires a huge amount of money and many parents take bank loans for which they have to pay a study interest, and the instruments, books used during the academic tenure also costs very high.

For clinical phase students, lack of co-operation by patients, Patients coming late or not coming for appointments, and getting an ideal case for clinical examination was severely stressful. Similar findings were observed in other studies as well.^12-14^ This may be due to the fact that to be eligible for the final examination, dental students are required to complete a certain quota of cases.

Lack of confidence in clinical decision making 59 (33%) was moderately stressful which is similar to the study conducted by Grewal et al.^14^ This may be due to exposure to limited cases and few skills in treating patients.

The main limitation of the study is that it was conducted among undergraduate students ofasingle dental institute which cannot be generalized to all undergraduate dental students in Nepal. Since the fourth domain is for clinical phase students only, stress level due to clinical factors is evaluated among the clinical phase only.

## CONCLUSIONS

The main stressors for the students in the present study were fear of failing, examination and grades, unable to catch up with back lock work, and patients related issues.

There is an urgent need of teaching various stress management techniques and positive coping strategies to deal with the demanding professional course by including stress management education in the curriculum.
